# Clinical-Pathological Conference Series from the Medical University of Graz: Case No 169: A 32-year-old woman with anemia in pregnancy

**DOI:** 10.1007/s00508-020-01679-w

**Published:** 2020-05-28

**Authors:** Elisabeth Fabian, Christoph Tinchon, Andreas Lueger, Philipp K. Bauer, Karoline I. Mayer-Pickel, Reinhold B. Raggam, Heinz F. Hammer, Cord Langner, Guenter J. Krejs

**Affiliations:** 1 Division of Gastroenterology and Hepatology, Department of Internal Medicine III, Medical University of Vienna, Vienna, Austria; 2 Division of Hematology and Oncology, Department of Internal Medicine, State Hospital Hochsteiermark, Leoben, Austria; 3 Division of Emergency Medicine, Department of Internal Medicine, Medical University of Graz, Graz, Austria; 4 Division of Infectious Diseases and Tropical Medicine, Department of Internal Medicine I, Medical University of Vienna, Vienna, Austria; 5 Department of Gynecology and Obstetrics, Medical University of Graz, Graz, Austria; 6 Division of Angiology, Department of Internal Medicine, Medical University of Graz, Graz, Austria; 7 Division of Gastroenterology and Hepatology, Department of Internal Medicine, Medical University of Graz, Auenbruggerplatz 15, 8036 Graz, Austria; 8 Department of Pathology, Medical University of Graz, Graz, Austria

**Keywords:** Celiac disease, Iron deficiency anemia, Vitamin D deficiency, Vitamin K deficiency, Hyposplenism

## Presentation of case

### Dr. A. Lueger:

The patient came to Austria as a refugee during the Yugoslav Wars 25 years ago. She was in the 7th week of pregnancy when her husband brought her to the emergency room (ER) of the Graz University Medical Center at 3 o’clock in the morning due to increasing dyspnea. She also complained of a dry cough but there was no fever. She had had a miscarriage 7 months previously and 3 years earlier, the patient had had a cesarean section at 40 weeks of gestation due to fetal distress. A healthy infant was delivered, weighing 3290 g and measuring 52 cm in length. At that time the hemoglobin before the cesarean section was 11.0 g/dL (normal: 12.0–15.3 g/dL), the mean corpuscular volume (MCV) was 82.8 fL (normal: 80–98 fL) and hemoglobin after delivery was 9.2 g/dL. On the current admission, she did not complain of any pain and there was no history of a vaginal discharge. She had no known allergies, and the only medication she took was oral replacement of folic acid and iron. Iron deficiency was said to have been present since youth but was never investigated. The electronic hospital record system also showed that she had come to the ER 16 months prior to admission because of fatigue, lassitude and exhaustion. The hemoglobin then was 10.4 g/dL, serum iron was 16 µg/dL (normal: 50–160 µg/dL) and ferritin 6 ng/mL (normal: 30–150 ng/mL). She received intravenous iron, and a work-up of the anemia was recommended, but not pursued by the patient. Physical examination revealed a slimly built person (55 kg, 170 cm, body mass index, BMI 19.0 kg/m^2^), blood pressure 110/80 mm Hg, heart rate 140 bpm, temperature 37.2 °C, O_2_ saturation 100% in room air and pale skin. There was no pulmonary or cardiac abnormality. Electrocardiogram showed sinus tachycardia. Laboratory results: hemoglobin 8.3 g/dL, MCV 60.3 fL, reticulocytes 13.9 ‰ (normal: 5–20 ‰), leukocytes 9.6 × 10^9^/L (normal: 4.4–11.3 × 10^9^/L), platelets 322 × 10^9^/L (normal: 140–440 × 10^9^/L), albumin 3.1 g/dL (normal: 3.5–5.3 g/dL), serum iron 10 µg/dL, transferrin 1.73 g/L (normal: 2.0–3.6 g/L), transferrin saturation 4% (normal: 16–45%), ferritin 11 ng/mL, vitamin D 8.1 ng/mL (normal: 30–60 ng/mL), parathyroid hormone 117.3 pg/mL (normal: 15–65 pg/mL), osteocalcin 98.0 ng/mL (normal: 1.0–35.0 ng/mL), prothrombin time 14% (normal: 70–120%), international normalized ratio (INR) 4.73 (normal: 2–3.5), activated partial thromboplastin time (APTT) 88.9 s (normal: 26–36 s), fibrinogen 606 mg/dL (normal: 210–400 mg/dL), antithrombin (AT) 108% (normal: >75%), D‑dimer 0.67 mg/dL (normal: <50 mg/dL), factor II 20% (normal: >70%), factor V 102% (normal: >70%), factor XI 59% (normal: >50%), factor XII 73% (normal: >50%), haptoglobin 2.84 g/L (normal: 0.3–2.0 g/L), folic acid 3.6 ng/mL (normal: 2.7–34.0 ng/mL), vitamin B_12_ 531 pg/mL (normal: 180–1100 pg/mL), C‑reactive protein (CRP) 62.4 mg/dL (normal: <5.0 mg/dL), thyroid-stimulating hormone 0.62 µU/mL (normal: 0.27–4.2 µU/mL). The patient received intravenous iron and vitamins K and D. Her levels of hemoglobin (12.8 mg/dL), MCV (85.5 fL) and ferritin (38 ng/mL) normalized within 3 weeks and so did prothrombin time and the serum vitamin D level. The remainder of the pregnancy was uneventful.

A diagnostic test was performed.

## Differential diagnosis

### Dr. C. Tinchon:

The patient under discussion is a young woman with increasing dyspnea and anemia in the 7th week of pregnancy. Anemia is a common condition in pregnancy and is defined as a hemoglobin level <11 g/dL in the first trimester and below 10.5 g/dL in the second and third trimester [1–3]. It affects up to 40% of pregnant women worldwide and nearly one third in the USA [4, 5]. It is a risk factor associated with antepartum, intrapartum and postpartum maternal morbidity, and perinatal morbidity and mortality with adverse effects arising proportionally to the severity of anemia [6]. The topic of anemia in pregnancy is complex and there is a wide variety of potential underlying etiologies that should be considered (Table 1). However, physiologic anemia (dilutional) and iron deficiency are the two most common causes of anemia in pregnant women. Physiologic anemia is due to an increase in plasma volume by 10–15% at weeks 6–12 of gestation with a further rapid expansion by weeks 30–34, and plateauing or slightly decreasing toward term. All of this occurs despite an adequate increase in red blood cell mass. At term, the total plasma volume of 4700–5200 mL is about 30–50% above that in non-pregnant women, while the increase in red cell mass is only about 10–30% [7]. This physiologic fall in hemoglobin concentration across pregnancy is often quoted as 5 g/L, but it was found to be as high as 8 g/L in some studies [8]. Insufficient supply of iron and micronutrients, such as folate, vitamins B_12_, B_6_ and copper due to inappropriate intake, hyperemesis gravidarum or malabsorption may also contribute to an imbalance between availability and increased nutrient requirements for fetal red blood cell production and fetoplacental growth, and consequently result in anemia. Iron deficiency is the most common cause of anemia worldwide and affects about one in three pregnant women [9, 10]. Laboratory data of the discussed patient showed significantly reduced levels of all iron status parameters (serum iron, transferrin saturation and ferritin) and confirmed the diagnosis of iron deficiency. However, this condition has been known for many years in this patient and could not be improved by regular oral iron supplementation. Assessment of iron status is sometimes challenging. Thus, the markedly reduced transferrin levels observed in the discussed patient may hint at malnutrition, protein deficiency, zinc deficiency, chronic liver injury or conditions, such as acute phase reactions, chronic disease or hereditary hypotransferrinemia [11]. Iron deficiency may be due to increased loss (gastrointestinal or urogenital bleeding, epistaxis, blood donation, hemoglobinuria caused by intravascular hemolysis), malnutrition, malabsorption, relative iron deficiency during therapy with erythropoietin, infection with *Helicobacter pylori*, or increased physiologic needs. Microcytic anemia may further be caused by chronic inflammation with a subsequent increase in hepcidin [12], which is an acute phase protein and hormone, and as such is a key regulator of systemic iron balance [13]. It inhibits iron entry into the plasma compartment from the three main sources of iron, i.e. dietary absorption in the duodenum, the release of recycled iron from macrophages and the release of stored iron from hepatocytes [13, 14]. Less frequently, microcytic hypochromic anemia may be sideroblastic (hereditary or acquired due to deficiencies in vitamin B_6_ or copper, or due to lead poisoning) and in rare cases it may be caused by thalassemia, sickle cell disease, or it may present as inherited atypical form of microcytic anemia. In the presence of fragmentocytes, microcytic hypochromic anemia may be artificial, i.e. resulting from hyponatremia.

**Table 1:** Causes of different forms of anemia in adults. (Adapted from [7])

Reticulocyte count	Microcytic MCV <80 fL	Normocytic MCV 80–100 fL	Macrocytic MCV >100 fL
Low or normal	Iron deficiency Anemia of chronic disease/inflammation Sideroblastic anemia Copper deficiency Vitamin B_6_ deficiency Zinc deficiency	Bleeding (acute) Iron deficiency (early) Anemia of chronic disease/inflammation Bone marrow suppression (cancer, aplastic anemia, infection) Chronic renal insufficiency Hypothyroidism Hypopituitarism Excess alcohol	Vitamin B_12_ or folate deficiency Excess alcohol Myelodysplastic syndrome Liver disease Hypothyroidism HIV infection Medications that interfere with nuclear maturation
Increased	Thalassemia Hemolysis	Bleeding (with bone marrow recovery) Hemolysis Bone marrow recovery (e.g. after infection, vitamin B_12_ or folate replacement and/or iron replacement)	Hemolysis Bone marrow recovery (e.g. after infection, vitamin B_12_ or folate replacement and/or iron replacement)

*MCV* mean corpuscular volume

Other causes of anemia besides physiologic factors and iron deficiency that are much less common but should be mentioned in the context of pregnancy include hemolytic anemia due to HELLP (hemolysis, elevated liver enzymes, low platelet count) syndrome, thrombotic thrombocytopenic purpura (TTP) and hemolytic uremic syndrome (HUS); however, in the discussed patient these diagnoses can be ruled out because of lacking clinical and laboratory findings suggesting these conditions (Table 2).

**Table 2:** Comparison of TTP, HUS and HELLP syndrome with regard to symptoms and laboratory findings [15, 16]

	TTP	HUS	HELLP
Abdominal pain	++	++	++
Low ADAMTS-13 activity	+/++	–	−/+
Anemia	++	++	+
Elevated LDH	++ very high values	++ very high values	++
Elevated transaminases	−/+	−/+	++
Fever	+	–	–
Headache or visual disturbance	++	–	++
Hypertension	+/++	++	++
Jaundice	–	–	+
Nausea and vomiting	++	++	++
Proteinuria	+ and hematuria	++	++
Thrombocytopenia	++	++	++
Von Willebrand factor	++	++	–

*TTP* thrombotic thrombocytopenic purpura, *HUS* hemolytic uremic syndrome, *HELLP* hemolysis, elevated liver function tests, low platelets, *LDH* lactate dehydrogenase, *ADAMTS-13* a disintegrin and metalloprotease with thrombospondin type 1 motif 13, +: prevalence of finding in affected patients

Laboratory data further revealed severe vitamin D deficiency with subsequently increased levels of parathyroid hormone and osteocalcin, and deficiency in vitamin K reflected by low levels of clotting factors II, VII, IX and X, predisposing to bruising and hemorrhage. Besides deficiencies in iron, vitamins D and K, the discussed patient also presented with low levels of albumin and transferrin, suggesting a deficiency in protein. Together with a low BMI, all these findings strongly hint at malnutrition or malassimilation, i.e. maldigestion or malabsorption. While maldigestion is due to reduced hydrolysis of nutrients (luminal or at the brush border level), malabsorption may be caused by (1) luminal factors (e.g. impaired hydrolysis, impaired micelle formation, limited bioavailability, bacterial overgrowth), (2) mucosal factors (impaired brush border hydrolase activity, inherited deficiencies, damaged absorbing surface, decreased absorbing surface, surgery, infiltration or infection), or (3) postabsorptive factors (e.g. obstruction of lymphatic system, deficiency in chylomicrons or beta-lipoproteins, protein-losing enteropathy) [17]. However, chronic iron deficiency and low serum folate levels despite regular supplementation strongly suggest a malabsorption problem in this patient.

Since the patient presented without diarrhea (silent malabsorption), we can exclude liver diseases with disturbed enterohepatic bile acid circulation as a differential diagnosis. Due to a negative history, we can rule out drugs that may cause diarrhea (e.g. olmesartan [18], mycophenolate mofetil, colchicine, cholestyramine, neomycin, calcium carbonate), tropical sprue (negative travel history), severe malabsorption syndromes in childhood (e.g. disaccharidase deficiency, Hartnup’s disease, cystinuria, acrodermatitis enteropathica, alpha-beta-lipoproteinemia, cystic fibrosis), surgery of the gastrointestinal tract (e.g. short bowel syndrome, bypass, fistulas), radiation (radiation enteritis), allotransplantation, inactivation of pancreatic enzymes due to Zollinger-Ellison syndrome, immunodeficiency syndromes and isolated fat malabsorption syndromes, such as benign familial hypobetalipoproteinemia. Because of lacking clinical symptoms and laboratory findings, chronic pancreatitis (which causes abdominal pain in up to 80% of patients and maldigestion in about 20% of patients, but not iron deficiency anemia), intestinal lymphoma, chronic merenteric ischemia and vasculitis can be excluded as a diagnosis in this pregnant patient. Since the patient did not complain of diarrhea, conditions such as a neuroendocrine tumor, infection with amoeba, *Trichuris trichiura* (whipworm) or cryptosporidium parasites, autoimmune enteropathy, intestinal amyloidosis, mastocytosis and non-celiac gluten sensitivity are unlikely in this case. Moreover, the absence of edema rules out protein-losing enteropathy as the underlying pathology, while absent eosinophilia makes eosinophilic gastroenteritis or infestation with hookworms *Strongyloides stercoralis, Paragonimus* spp. or *Schistosoma *spp. an improbable diagnosis [19]. As a differential diagnosis of malabsorption, Whipple‘s disease, a rare chronic infection in which almost all organ systems can be invaded by the rod-shaped bacterium *Tropheryma whipplei*, should be considered as well. *Tropheryma whipplei* is a ubiquitous bacterium with a wide clinical spectrum of infections encompassing chronic systemic infection (classical Whipple’s disease), chronic focal infections, acute infections and healthy carriage [20]. The annual incidence in central European countries is estimated to be approximately 1/1,000,000 [21]. It primarily affects middle-aged white men. The main clinical features include gastrointestinal symptoms, such as diarrhea and abdominal pain, joint symptoms and weight loss. Up to 60% of affected patients complain of recurrent arthritis, and up to 40% suffer from sacroiliitis [22]. Furthermore, involvement of the central nervous system, cardiac manifestations (endocarditis) and pulmonary infiltration have all been described [23]. Although the laboratory data showed a markedly increased level of CRP, which would also be typical for Whipple’s disease, the clinical features of the patient did not suggest this rare disorder. In addition, small intestinal bacterial overgrowth and infection with *Diphyllobothrium latum* can be ruled out in this case because both conditions would go along with vitamin B_12_ deficiency, which was not present in our patient.

Once considered a pediatric problem, celiac disease has now become an important differential diagnosis in adults as well. Celiac disease, also known as celiac sprue, nontropical sprue or gluten-sensitive enteropathy, is a chronic enteropathy characterized by an autoimmune response in genetically susceptible individuals that affects people of all ages worldwide [24]. In western countries, the prevalence of celiac disease is about 1% of the general population [25, 26]. Classical celiac disease diagnosed in children typically presents with diarrhea, malabsorption, failure to thrive and growth retardation [27]. In adults, the clinical presentation of celiac disease can vary from the asymptomatic state to malabsorption, micronutrient deficiencies, osteoporosis and neurological disorders ([28]; Table 3). Due to malabsorption of micronutrients, anemia and osteopenia or osteoporosis can most often be found in patients with newly diagnosed celiac disease. Anemia, usually secondary to iron deficiency and often refractory to oral iron treatment, affects 60–80% of newly diagnosed patients [29–31], and about 75% of patients have some degree of bone loss [32–34]. Therefore, it is recommended to obtain celiac antibodies whenever there is a clinical or biochemical suspicion of malabsorption [35]. Serological testing includes anti-tissue transglutaminase (tTG-IgA) antibodies and anti-endomysial antibodies (EmA-IgA), detected by immunofluorescence, with equivalent diagnostic accuracy. The anti-gliadin antibody (AGA) test is less reliable; however, it is suggested that IgG and IgA antibodies against deamidated gliadin peptides (DGP)-AGA have a comparable diagnostic accuracy as tTG-IgA [36, 37]. An IgA deficiency is about 10–15 times more common in patients with celiac disease than in healthy individuals. Genetic testing of HLA-DQ2 and HLA-DQ8 is not an absolute requirement for diagnosis, but a negative result makes celiac disease unlikely. In Europe, 85–90% of patients with celiac disease are positive for HLA-DQ2, and 10–15% are positive for HLA-DQ8 [38]. However, it should be considered that 30–40% of the general population are also positive for these alleles (with HLA-DQ2 more common than HLA-DQ8) but do not have the disease [39]. HLA testing needs to be performed only once during the lifetime, initial negative serological tests, however, do not exclude the development of celiac disease later in life. Histopathological changes are characterized by typical architectural abnormalities as defined by the Marsh-Oberhuber classification ([40]; Table 4). Although gluten-free diet usually results in good clinical response, abnormal histopathological findings persist in a high percentage of patients [41, 42]. Nevertheless, for the diagnosis of celiac disease, it is important that serological and histological diagnostic tests are performed while the patient is on a gluten-containing diet because otherwise the tests may be inconclusive and necessitate a gluten challenge.

**Table 3:** Different presentations of celiac disease in adults. (Adapted from [24, 53])

	Asymtomatic		Symptomatic	
	Latent	Silent	Atypical	Classic
Villous atrophy^a^	−	+	+	+
Serology (tTG-IgA)^a^	+	+	+	+
HLA-DQ2 or DQ8	+	+	+	+
Nonspecific symptoms^b^	−	−	+	+/−
Chronic diarrhea, steatorrhea	−	−	−	+

^a^Not all patients are positive at presentation; this might be due to IgA deficiency or gluten-free diet for more than 3 months prior to testing

^b^e.g. iron deficiency anemia, osteoporosis, irritable bowel syndrome

**Table 4:** Marsh-Oberhuber classification of architectural abnormalities in celiac disease [40]

Marsh	0	1	2	3a	3b	3c
IEL/100 epithelial cells	≤25	>25	>25	>25	>25	>25
Crypts	Normal	Normal	Hyperplasia	Hyperplasia	Hyperplasia	Hyperplasia
Villus	Normal	Normal	Normal	Moderate atrophy	Subtotal atrophy	Total atrophy
Type of lesion	Remission	Infiltrative	Hyperplastic	Destructive	–	–

*IEL* intraepithelial lymphocytes

An entity that is less frequently observed in western countries but should nevertheless be mentioned as a differential diagnosis in this case, is infection with *Giardia lamblia*. This protozoan parasite attaches to the intestinal epithelium in the duodenum and jejunum, and disrupts the epithelial barrier function by altering tight junction composition and increasing apoptosis [43]. Symptoms of acute infection include abdominal pain, diarrhea, bloating and greasy stools that tend to float; indigestion or nausea and vomiting are also frequently reported [44–46]. In chronic giardiasis, symptoms fluctuate and steatorrhea may be due to the formation of a layer of *Giardia* trophozoites that are attached to the duodenal mucosa by a large ventral sucking disk. Besides the described cellular and mechanical effects resulting in increased epithelial permeability, *Giardia lamblia* also causes intestinal abnormalities in the host, such as the loss of intestinal brush border surface area and villus flattening, similar to that observed in celiac disease [47]. Consequently, infection with *Giardia lamblia* can lead to malabsorption which in rare cases may result in vitamin K deficiency and impaired coagulation [48] as observed in the discussed patient. However, since eosinophilia is often present in infections with* Giardia lamblia* but was not present in this case, giardiasis can probably be ruled out as a diagnosis.

Due to evidence of malabsorption in this patient, the differential diagnosis should also include Crohn’s disease, which can involve all parts of the gastrointestinal tract and present with increased CRP levels (as found in the discussed patient), nausea, vomiting and epigastric pain [49–51]. Although Crohn’s disease usually afflicts patients in their 20s and 30s, exacerbation during pregnancy is typically seen in the 2nd or 3rd trimester, but not in the 1st trimester as in the discussed patient. Other forms of inflammatory bowel disease such as ulcerative colitis and indeterminate colitis, predominantly involving the rectum and variable parts of the colon, are usually not associated with malabsorption and are thus unlikely diagnoses in this case.

All these considerations finally lead to the suggested diagnostic approach of (1) endoscopy with duodenal biopsies, (2) serological testing for tTG-IgA antibodies, (3) quantitative analysis of immunoglobulins and Ig subtypes to rule out IgA deficiency, and (4) testing for parasites and ova in stool, or duodenal aspirate analysis for exclusion of giardiasis.

## Dr. C. Tinchon’s diagnosis

Celiac disease

## Discussion of case

### Dr. A. Lueger:

As the patient’s attending physician, I highly suspected a malabsorption syndrome when routine laboratory data revealed low levels or deficiencies in several micronutrients despite the reported regular supplementation of iron and folate. Since celiac disease is the most frequently occurring malabsorption syndrome, we obtained a measurement of serum tTG-IgA antibodies, and the initial value was 716 U/mL (normal: up to 16 U/mL). The patient was put on a gluten-free diet, and after initial parenteral replacement followed by oral replacement therapy (iron, vitamins D and K), all abnormal parameters returned to normal and they remained unchanged on continued gluten-free diet alone. After 6 months, the tTG-IgA antibody level dropped to the normal range at 15 U/mL.

### Dr. G. J. Krejs:

Celiac disease is an immune-mediated enteropathy with a strong genetic predisposition, in most cases improving on dietary exclusion of gluten [52]. Thus, for the diagnosis, it is important that diagnostic work-up is performed while the patient is on a gluten-containing diet, because otherwise the results may be inconclusive. As mentioned, intestinal mucosal biopsy is the gold standard for diagnosis. In the discussed patient, however, endoscopy was not performed because of pregnancy. What is the pathologist’s opinion on a diagnosis of celiac disease without a small bowel biopsy?

### Dr. C. Langner:

According to the consensus of the German Society of Gastroenterology, Digestive and Metabolic Diseases and the German Celiac Society, celiac disease can be diagnosed in patients with positive serology and positive histology (i.e. Marsh 2 or 3), and improvement of serological markers on gluten-free diet. Biopsy is not necessary in children with clinical symptoms and signs of malabsorption, who have a serum tTG-IgA antibody titer >10 times the upper reference limit, positive EmA-IgA antibodies (second independent sample), are positive for HLA-DQ2 or HLA-DQ8 and clinically improve on a gluten-free diet [53]. For histological work-up, at least 6 biopsies should be obtained from different parts of the duodenum including the duodenal bulb, the middle and distal duodenum. Celiac disease is characterized by specific histopathological changes including partial or total villous atrophy, crypt hyperplasia, an altered villus to crypt ratio, an increase in intraepithelial lymphocytes (IEL), and increased infiltration of the lamina propria with plasma cells, lymphocytes and eosinophilic and basophilic granulocytes [40]. The typical architectural abnormalities are defined by the Marsh-Oberhuber classification (Table 4). In patients who clinically do not respond to a gluten-free diet, a repeat biopsy is recommended to verify refractory celiac disease type I or type II. Data show that a gluten-free diet results in good recovery to normal mucosal architecture in about 96% of patients after 2 years. Only 4% of patients display a persistently abnormal mucosal architecture (Marsh 2 or 3). However, the number of IELs is normal in only 56%, and pathologic in 44% of patients with recovered villous architecture [54]. Regarding the duration of gluten-free diet, it is observed that with time (2–5 years, 5–10, 10–15, 15–20 and over 20 years), persistence of IELs dropped to 85%, 63%, 51%, 48% and 48%, respectively. Lowering the cut-off value for IELs to 25 IEL/100 epithelial cells resulted in an increase of this histopathological finding to 89% of patients after 2–5 years on a gluten-free diet, and to 67% after >20 years [54]. Thus, persistence of intraepithelial lymphocytosis is not an indicator of refractory celiac disease. For diagnosis of celiac disease, mucosal architecture (i.e. the appearance of villi and crypts) and clinical symptoms are relevant. In the discussed case, esophagogastroduodenoscopy (EGD) with biopsies was not performed because the patient was pregnant and became totally free of symptoms on a gluten-free diet. Thus, EGD does not seem to be necessary in this patient at this time but could be done to verify the histopathological features on a gluten-free diet later.

### Dr. G. J. Krejs:

Given the dramatic drop in tTG-IgA antibodies and the spectacular response to the gluten-free diet, we think that the diagnosis of celiac disease is conclusive in this case. A small bowel biopsy would be of academic interest to see whether the mucosa has returned to normal, or how much residual disease has remained as described by Dr. Langner.

### Dr. K. I. Mayer-Pickel:

On admission, transvaginal sonography of the pregnant patient (7th week of gestation) showed a normally developed, 8 cm fetus with positive heart action. Besides an intrauterine scar from a former cesarean section, sonography revealed distended small bowel segments with moderate movements in the lower quadrants. Since the further course of the patient’s pregnancy was unremarkable, routine medical care of the mother and the fetus was provided by her local gynecologist. In the 37th week of gestation, she was seen again in the outpatient clinic for the purpose of planning an elective cesarean section. At that time, the patient reported to be free of gastrointestinal complaints on a gluten-free diet. Laboratory data showed normal hemoglobin (13.7 g/dL, MCV 86.4 fL) and prothrombin time (110%). One week later, the patient gave birth to a healthy boy (body weight 3450 g, APGAR score 6/8/10) without complications. Thus, the mother and child were discharged on the third postpartum day.

### Dr. G. J. Krejs:

As reflected by the laboratory data, micronutrient deficiencies were not observed any longer when the patient was on a gluten-free diet. However, if you look at the chances of having celiac disease from the angle of iron deficiency anemia, a recent systematic review showed that 1 in 31 patients with iron deficiency anemia is found to have celiac disease [55]. As in the discussed patient, vitamin K deficiency reflected by disturbed coagulation is also frequently observed in celiac disease. However, at term, coagulation had normalized upon instituting substitution and elimination of malabsorption by the gluten-free diet. Dr. Raggam, who is an expert in the field of coagulation, was consulted in this case and will now comment.

### Dr. R. B. Raggam:

On admission of the discussed patient, both global tests for coagulation, i.e. APTT (88.9 s) and prothrombin time (14%), were significantly altered and the serum level of fibrinogen was markedly increased (606 mg/dL). The APTT and prothrombin time cover all clotting factors except factor XIII and are useful to get an impression of the coagulation system. While a prolonged APTT primarily reflects low levels of clotting factors XII, XI, IX and VIII, it will also be increased when factors X, V or fibrinogen are deficient. Prothrombin time indicates availability of vitamin K-dependent clotting factors (VII, X, II), but it will also be altered when the levels of factor V or fibrinogen are low. The alterations observed in the present case strongly suggest vitamin K deficiency, which can be due to low dietary intake or malabsorption, or it may be iatrogenic due to treatment with warfarin, superwarfarin or antibiotics. However, the differential diagnosis should also include the nonspecific (antiphospholipids or lupus inhibitor) and specific acquired antibodies against coagulation factors, hyperfibrinolysis or disseminated intravascular coagulation (DIC). Since our patient had an increased level of fibrinogen, normal thrombocytes and a physiologic increase in D-dimer, DIC could be ruled out. On clinical examination, the patient did not show signs of bleeding, which suggests a slowly developing coagulation disturbance with a consequent adaption of procoagulant factors, i.e. factor VIII, von Willebrand factor and fibrinogen. To further differentiate between deficiency in coagulation factors and acquired inhibitors of coagulation factors, the plasma mixing test was employed. This test resulted in a near-normal APTT (42.5 s) and a normal prothrombin time (71%), and thus clearly indicated coagulation factor deficiency in our patient. To evaluate the bleeding risk, thromboelastography, which is a graphical presentation of the formed clot that allows a rapid identification of the underlying cause of disturbed coagulation (i.e. deficiency in coagulation factors, platelets or fibrinogen), was performed. While the clotting time reflects APTT and prothrombin time in this test, the α‑angle, the maximum amplitude of the formed clot and lysis time are indicators of clot formation and stability. In the discussed patient, thromboelastography clearly identified a deficiency in coagulation factors, but it did not indicate an increased bleeding risk as reflected by a steep α‑angle of the formed clot and high clot stability without lysis. The results of thromboelastography show that substitution with coagulation factors was not indicated in the absence of bleeding and may even predispose the patient to thrombotic events. Analysis of single coagulation factor activities then revealed a deficiency in vitamin K-dependent factors II, VII, IX and X.

### Dr. G. J. Krejs:

Among 174 clinical-pathological conferences in this institution in the last 33 years, 4 cases of celiac disease have been discussed. Celiac disease in adults remains a challenging diagnosis since the clinical presentations can be so different. Dr. Hammer is in charge of the outpatient care that we offer adults with celiac disease.

### Dr. H. Hammer:

Celiac disease is a chronic multiorgan autoimmune disease that affects the small intestine in genetically predisposed persons, precipitated by the ingestion of gluten [56, 57]. The disease affects persons from diverse ethnic backgrounds. In western countries, the prevalence of histologically confirmed celiac disease is around 0.6%, and 1% in serological screening of the general population [58]. A large proportion of patients are diagnosed above the age of 20 years, and while some of these patients may probably have had undetected disease since childhood, other patients developed the disease in adulthood [59]. A subgroup of patients is regarded as potential or latent celiac disease because they have a normal small bowel mucosa but positive serology along with a positive HLA status (DQ2 or DQ8) [60]. From my point of view as a clinical gastroenterologist, iron deficiency is a typical presentation of celiac disease, especially if, as in this case, it has persisted for many years, even in the face of ongoing oral iron substitution, or if it is accompanied by other signs of malnutrition and consequences of malabsorption, which have been extensively discussed by the previous speakers and to which I would like to add problems with previous pregnancies. More recently, celiac disease is also suspected and recognized in patients with symptoms resembling the irritable bowel syndrome, osteopenia, amenorrhea, and even small bowel lymphoma. According to the most recent guidelines, the diagnosis of celiac disease in adults is based on a combination of clinical, serological and histopathological data [60], and tests should be performed while the patient is on a gluten-containing diet. Histology alone is not sufficient for the diagnosis as there are many histological mimics of celiac disease in seronegative patients [60]. The indications for testing for celiac disease are shown in Table 5.

**Table 5:** Recommendations for testing for celiac disease in adults according to the guidelines of the European Society for the Study of Coeliac Disease (ESsCD) [60]

Endoscopy and duodenal biopsy even if serology is negative	Serology is indicated: biopsy is needed only when serology is positive
Chronic (non-bloody) diarrhea Diarrhea with features of malabsorption, especially weight loss Iron deficiency anemia in absence of other causes Gastrointestinal symptoms with a family history of celiac disease Gastrointestinal symptoms in patients with an autoimmune disease or IgA deficiency Skin biopsy proven dermatitis herpetiformis Patient with video capsule findings suggestive of villous atrophy Unexplained high-output ileo(colo)stomy	Irritable bowel syndrome Elevated, otherwise unexplained liver transaminases Chronic gastrointestinal symptoms without a family history of celiac disease or a personal history of autoimmune disease Microscopic colitis Hashimoto thyroiditis and Graves’ disease Osteopenia, osteoporosis Unexplained ataxia or peripheral neuropathy Recurrent aphthous ulcers or dental enamel defects Infertility, recurrent miscarriage, late menarche, early menopause Chronic fatigue syndrome Acute or chronic pancreatitis, after excluding other causes Epilepsy, headaches including migraines, mood disorders or attention deficit disorder, cognitive impairment Hyposplenism or functional asplenia Psoriasis or skin lesions other than dermatitis herpetiformis Down’s or Turner’s syndrome Pulmonary hemosiderosis IgA nephropathy

### Dr. G. J. Krejs:

In this case, both the mother and child tested positive for HLA-DQ2. We asked two independent mathematicians about the risk faced by a newborn to contract celiac disease in the future. Based on the prevalence of celiac disease of 1% in the general population and the fact that 90% of patients are HLA-DQ2 positive as compared to only 10% of the general population, calculation revealed a risk of 7.4% for the newborn to develop celiac disease in the future. The pediatrician taking care of the child should consider this when regularly screening the patient to ensure early detection and treatment if necessary.

When I came to the Medical University of Graz 33 years ago, celiac disease was primarily diagnosed in children, but hardly recognized in adults which often led to delays in diagnosis and treatment of the affected patients. For instance, we had a 60-year-old patient who was suspected to have metastatic prostate cancer, but the bone changes were due to celiac disease which was diagnosed as late as during his 12th hospital admission.

Celiac disease is a disorder associated with splenic hypofunction or atrophy (hyposplenism), affecting about one third of adult patients. This may predispose to a higher risk of infections and other complications, as explained by Dr. Bauer.

### Dr. P. K. Bauer:

Hyposplenism, which occurs in 25–75% of patients with celiac disease [61], is a common conditon that may result in increased risk of invasive infection caused by encapsulated bacteria, and further predisposes to autoimmune diseases and thromboembolic complications. The spleen is pivotal in the regulation of immune homeostasis by linking innate and adaptive immunity and protecting against infections [62, 63]. Impaired function subsequently leads to (1) a reduced number of IgM memory B cells and defective activity of opsonizing molecules, which facilitates infections by encapsulated bacteria, such as *Streptococcus pneumoniae, Neisseria meningitidis* and *Haemophilus influenzae*, and (2) a decreased number of marginal zone B cells, which predisposes to the development of autoimmunity due to regulatory T cell depletion and subsequent increase in autoreactive T cell clones [64]. Thus, hyposplenic individuals are vulnerable to invasive infections in particular with *Streptococcus pneumoniae,* which is responsible for up to 50% of such infections and often presents with a sudden onset and a fulminant course [65]. Hyposplenic adult patients with celiac disease face a higher risk of respiratory diseases (mainly pneumonia) [66, 75] and pneumococcal sepsis [67, 68]. However, the incidence of infection can be reduced by preventive measures such as vaccination. Currently, a 23-valent pneumococcal polysaccharide vaccine exerting its protective effect via a T cell-independent mechanism is recommended for asplenic or hyposplenic adults and children over 5 years of age, and a 13-valent protein conjugate pneumococcal vaccine acting via a T cell-dependent mechanism is available for asplenic or hyposplenic children <5 years [64]. In patients with celiac disease the immunological response to vaccination is the same as in the general population [76].

Further, spleen function was found to be crucial for the presence of IgA-producing plasma cells in the gut [69] and maintenance of oral tolerance to gluten [70]. Consequently, the incidence of hyposplenism correlates with the duration of pre-exposure to gluten as shown by the correlation with age at diagnosis [71]. However, a gluten-free diet does not seem to have a positive effect on the development of hyposplenism in adult patients with celiac disease [72].

Besides immunological consequences of hyposplenism, the filtering function of the spleen is also impaired in this condition. This results in (1) reduced platelet sequestration which is associated with increased risk of thromboembolism and (2) inappropriate removal of pits from erythrocytes increasing circulating Howell-Jolly bodies and pitted red cells, which in turn predisposes to hyperviscosity [73]. Thus, patients with celiac disease may also face an increased risk of thromboembolism. However, this risk may also be influenced by altered clotting factors and development of a procoagulative condition secondary to vitamin K deficiency, as observed in the discussed patient. According to the review article by Balaban et al. [74], extraintestinal manifestations of celiac disease are becoming increasingly prevalent as the initial presenting manifestation. Hematologic features of the disease are occurring quite frequently and can be the sole manifestation of celiac disease. Changes in platelet count or iron status can hint at celiac disease. Screening for celiac disease within this cohort of patients should be kept in mind as well as in patients with IgA deficiency or hemorrhagic manifestations, which cannot be explained otherwise.

## Final diagnosis

Celiac disease with malabsorption of iron, and vitamins D and K.
